# Human-Derived Corneal Epithelial Cells Expressing Cell Cycle Regulators as a New Resource for *in vitro* Ocular Toxicity Testing

**DOI:** 10.3389/fgene.2019.00587

**Published:** 2019-07-16

**Authors:** Tomokazu Fukuda, Ryo Gouko, Takahiro Eitsuka, Ryusei Suzuki, Kohei Takahashi, Kiyotaka Nakagawa, Eriko Sugano, Hiroshi Tomita, Tohru Kiyono

**Affiliations:** ^1^ Graduate School of Science and Engineering, Iwate University, Morioka, Japan; ^2^ Soft-Path Engineering Research Center (SPERC), Iwate University, Morioka, Japan; ^3^ Graduate School of Agricultural Science, Tohoku University, Aoba-ku, Sendai, Japan; ^4^ Division of Carcinogenesis and Cancer Prevention, National Cancer Center Research Institute, Tokyo, Japan

**Keywords:** corneal epithelial cells, immortalization, cell cycle regulators, cyclin-dependent kinase 4, cyclin D1, telomerase reverse transcriptase

## Abstract

The Draize test has been used on rabbits since the 1960s to evaluate the irritation caused by commercial chemicals in products such as cosmetics or hairdressing products. However, since 2003, such tests, including the Draize test for cosmetics, have been prohibited in European countries because they are considered problematic to animal welfare. For this reason, replacement of *in vivo* methods with the alternative *in vitro* methods has become an important goal. In this study, we established a corneal epithelial cell line co-expressing a mutant cyclin-dependent kinase 4 (*CDK4*), Cyclin D1, and telomerase reverse transcriptase (*TERT*). The established cell line maintained its original morphology and had an enhanced proliferation rate. Furthermore, the cells showed a significant, dose-dependent decrease in viability in an irritation test using glycolic acid and Benzalkonium chloride. These cells can now be shared with toxicology scientists and should contribute to increasing the reproducibility of chemical testing *in vitro*.

## Introduction

The safety of chemical compounds, especially the evaluation of their potential as irritants, is important for commercial chemicals that are used daily, such as cosmetics or hairdressing products. Since the 1960s, various types of animal experiments have been performed to evaluate the potential side effects of commercial chemicals (Buehler and Newman, 1964). As one representative animal experiment, the Draize test using rabbits has been a practical standard. However, animal experiments, including the Draize test for cosmetics, have been prohibited in European countries since 2003 because this method is currently considered problematic from the viewpoint of animal welfare (Sharpe, 1985). For this reason, the development of alternative *in vitro* ocular toxicity tests is worth further investigation.

Corneal epithelial cells exist at the outermost layer of the cornea and play a role in protecting the eyes from invasion by foreign materials. Corneal epithelial cells could, therefore, be useful for *in vitro* ocular toxicity testing. In this regard, an irritation test using rabbit corneal epithelial cells, referred to as the short time exposure (STE) test, has been previously established (Takahashi et al., 2008). However, since there is a species difference between humans and rabbits, the results of an irritation test that uses rabbit cells are likely to be different from what is actually experienced by the human eye. For this reason, we considered the use of human corneal epithelial cells as a new approach to *in vitro* ocular testing. However, primary corneal epithelial cells are limited in their usefulness because these cells stop growing after only a few passages. The halt of cell proliferation can mainly be attributed to cell culture stress and Hayflick limitation (Hayflick and Moorahead, 1961).

To overcome the limitations of cellular senescence, several standard methods to immortalize cells have been established. Simian vacuolating virus 40 (SV40) and E6/E7 human papillomavirus-derived oncoproteins are well known to inactivate p53 and retinoblastoma protein (RB) (Tsao et al., 2002) and are effective for standard cellular immortalization. However, immortalized cells using SV40 or E6/E7 are reported to have abnormalities in their chromosomes (Ray et al., 1990; Duensing et al., 2000).

In 2011, a newly developed method for cell immortalization was reported. Briefly, Shiomi et al. (2011) achieved the immortalization of primary cells by co-expressing a mutant (R24C) cyclin-dependent kinase 4 (*CDK4*), cyclin D1, and telomerase reverse transcriptase (*TERT*). The immortalized cells maintained the karyotype and differentiation ability of the original cells (Shiomi et al., 2011). Based on the names of the expressed cells, this established method was referred to as the K4DT method (mutant CDK4, Cyclin D, and TERT). Furthermore, our group reported that this newly established K4DT method could be used in a variety of species of animals, including bovine, swine, monkey, prairie vole, and midget buffalo, which could possibly be explained by the highly conserved amino acid sequence of these cell cycle regulators in these animals (Donai et al., 2014; Kuroda et al., 2015; Katayama et al., 2017). Based on these data, we formed the hypothesis that co-expression of mutant *CDK4*, *Cyclin D1*, and *TERT* might allow us to establish a new corneal epithelial cell line, which retains the original nature of the primary cells better than the traditional oncogenic method. In this study, we report the establishment of human corneal epithelium-derived cells and its biological characterization for toxicity evaluation. These cells should contribute to the evaluation of chemical toxicity with high reproducibility. Furthermore, these cells can now be shared with toxicology scientists, which should promote the replacement of animal models for experimentation and contribute to animal welfare.

## Materials and Methods

### Cell Culture

Corneal epithelial cells were commercially obtained from Lifeline Cell Technology (Frederick, MD, USA; cat. no. FC-0029) through Kurabo (Osaka, Japan). The cells were cultured in six-well dishes with OcuLife basal medium (Lifeline Cell Technology; cat. no. LM-0012) containing OcuLife LifeFactors (Lifeline Cell Technology; cat. no. LS-1057) with 6 mM L-glutamine, 0.4% v/v bovine pituitary extract, 1.0 μM epinephrine, 100 ng/ml hydrocortisone hemisuccinate, 5 μg/ml recombinant human insulin, and 5 μg/ml apo-transferrin at 37°C in an atmosphere of 5% CO_2_. Before cell passage, the cells were washed with 1 × D-PBS (phosphate buffered saline) (−) (Nacalai Tesque, Kyoto, Japan; cat. no. 11482-15) and dispersed with StemPro Accutase (Life Technologies, Waltham, MA, USA; cat. no. A11105-01) for 5 min at 37°C. The dispersed cells were then centrifuged at 800 × *g* for 5 min, and the pelleted cells were resuspended in fresh medium.

### Preparation of Recombinant Lentiviruses and Infection to the Cells

To establish the new corneal epithelial cell line, the primary cells were infected with recombinant lentiviruses. The basic backbone of the recombinant lentiviruses was derived from the CSII vector, which was kindly provided by Dr. Miyoshi (Keio University, Tokyo, Japan). CSII-CMV-CDK4R24CF2A-CyclinD-IRES (internal ribosomal entry site)–EGFP (enhanced green fluorescent protein) is a polycistronic vector that expresses both CDK4R24C and Cyclin D. In order to monitor transfection efficiency, the CSII-CMV-CDK4R24CF2A-CyclinD-IRES–EGFP was constructed such that the expression cassette was linked with EGFP through an IRES. Corneal epithelial cells were also infected with a mixture of three monocistronic lentiviruses, CSII-CMV-TERT, CSII-CMV-CyclinD, and CSII-CMV-hCDK4R24C. We named the corneal epithelial cells infected with polycistronic virus as K4D (CDK4R24C + Cyclin D) cells and the corneal epithelial cells infected with a mixture of monocistronic lentiviruses as K4DT cells (CDK4R24C + Cyclin D + TERT). Details of the production of these recombinant viruses and their infection have been described in our previous report (Fukuda et al., 2016). The titer of the TERT lentivirus was usually lower than that of the mutant CDK4 and Cyclin D lentiviruses, due to it having the relatively longer cDNA insert size of around 4 kb (Fukuda et al., 2018). Therefore, K4DT cells were additionally infected with a retrovirus, which expresses TERT and a hygromycin selection marker. We refer to these hygromycin-resistant cells as K4DT + T cells.

### Western Blotting

To extract proteins from primary, K4D, and K4DT + T cells, we lysed cells in a buffer containing 50 mM Tris–HCl (pH 7.4), 0.15 M NaCl, 1% Triton X-100, 2.5 mg/ml sodium deoxycholate (Wako, Osaka, Japan; cat. no. 194-08311), and a protease inhibitor cocktail (1/200 dilution, Nacalai Tesque; cat. no. 25955-11). The protein expression levels of CDK4 and Cyclin D were detected by Western blotting using an anti-CDK4 antibody (1/2,500 dilution, MBL, Nagoya, Japan; cat. no. 25955-11), an anti-cyclin D antibody (1/5,000 dilution, MB; cat. no. 553), and an anti-alpha-tubulin antibody (1/1,000 dilution, Santa Cruz Biotechnology, Dallas, TX, USA; cat. no. sc-32,293). Anti-mouse IgG (1/2,000 dilution, GE Healthcare, Buckinghamshire, UK; cat. no. NA931) or anti-rabbit IgG (1/2,000 dilution; GE Healthcare; cat. no. NA934-1ML) was used as secondary antibodies. The detailed Western blot procedure has been reported previously (Fukuda et al., 2005, 2008). The intensity of signals was measured by the Image J software.

### Population Doubling Assay

To measure the proliferative capacity of primary, K4D, and K4DT + T cells, we sequentially passaged the cells. Each cell line was initially seeded into six-well plates at a density of 5.0 × 10^4^ cells in triplicate. When the cell line reached confluency, the cells were dispersed using StemPro Accutase (Life Technologies). We recorded the total number of cells in each dish using an automatic cell counter (Thermo Fisher Scientific, Waltham, MA, USA). Cells (1.0 × 10^5^) were then seeded into a new dish to evaluate their growth rate by determining the population doubling level (PDL). PDL was calculated using the following equation: PDL = log_2_ (*A/B*), where *A* is the number of cells harvested at each passage and *B* is the number of seeded cells.

### PCR Analysis

We extracted genomic DNA from the primary, K4D, and K4DT + T cells using NucleoSpin Tissue (Takara Bio, Shiga, Japan; cat. no. 740952) following the manufacturer’s protocol. To monitor insertion of the transgene, TERT and Tuberous Sclerosis Type II (TSC2; internal control) were detected using a PCR analysis. TSC2 was chosen as the internal control, since the TSC2 gene is a unique gene in the human genome, and furthermore there are no TSC2 pseudogenes. The cDNAs were amplified in a reaction solution containing KOD FX Neo (Toyobo, Osaka, Japan; cat. no. KFX-201) dNTPs and specific primers. After amplification, the PCR reaction was mixed with loading dye, and the PCR products were separated by electrophoresis on 1% agarose gels followed by ethidium bromide staining.

### Fluorescent Staining of F-Actin

We seeded the primary, K4D, and K4DT + T cells into a Lab-Tek chamber Slide (Thermo Fisher Scientific) to determine the morphology of each cell line. The cells were exposed to 500 μl of 0.2% Triton X-100 to permeabilize them, after which rhodamine-labeled phalloidin (1/40 dilution, Wako, Osaka, Japan; cat. no. 165-21641) was used to stain F-actin, and the nuclei were counterstained with DAPI (4′,6-diamidino-2-phenylindole) (1/300 dilution, Dojindo, Kumamoto, Japan; cat. no. 28718-90-3). We captured fluorescence images with a benchtop microscope (Keyence, Osaka, Japan; cat. no. BZ-9000).

### Cell Cycle Analysis

For cell cycle analysis, approximately 4 × 10^5^ primary, K4D, and K4DT + T cells were fixed with 70% ethanol. Six replicates within each experimental group were stained with the cell cycle assay kit (Merck Millipore, Darmstadt, Germany; cat. no. MCH100106). The stained cells were then analyzed using a Muse cell analyzer (Merck Millipore). The results were statistically evaluated using a non-parametric multiple comparison test by the steel method.

### Immunostaining

For the immunostaining, we used Lab-Tek Chamber slide with Permanox Slide for the cell culture of corneal epithelial cells (Thermo Scientific Nunc, Waltham, MA USA). The cells were washed with 1× PBS and fixed with 4% paraformaldehyde solution (Nacalai Tesque). The cells were permeabilized with Triton X-100 solution, and blocking reaction was carried out with 1% bovine serum albumin in PBS. Primary antibody (anti-Cytokeratin 3/2p, Santa Cruz, sc-80,000) was exposed at 1:40 dilution with blocking buffer. The secondary antibody Alexa 568-labeled goat anti-mouse IgG was used for the detection with the counterstaining by DAPI. The staining images were obtained by a fluorescence microscope (BZ-9000, Keyence, Tokyo, Japan).

### Ocular Toxicity Test

We evaluated irritation based on the protocol for the STE test, as previously described (Takahashi et al., 2008). We selected glycolic acid (Wako; cat. no 071-01512) as a positive chemical for the irritant test. Briefly, primary cells or K4DT + T cells in six-well plates were exposed to either a 0.5% or 5% glycolic acid solution for 5 min; PBS was used as a negative control. Furthermore, we also tested the toxicity of Benzalkonium chloride. The cells in each well were washed twice with PBS and then treated with Accutase for 5 min. The total number of cells and the cell viability were determined using trypan blue staining. To avoid bias in cell counting, an automatic cell counter was used for all measurements. Cell viability was calculated based on the ratio of the number of living cells relative to the total cell number.

### MTS Assay

We detected cell toxicity of Benzalkonium chloride with MTS assay. MTS assay is a colorimetric method for determining the number of viable cells in proliferation, cytotoxicity, or chemosensitivity. The corneal epithelial cell immortalized with K4DT expression was seeded around 80% density in a 96-well plate with 100 μl of medium. We exposed the cells to 5, 0.5, 0.05, 0.005, and 0.0005% Benzalkonium chloride solution in PBS. After the 5-min exposure to the cells, the medium was changed into basal medium and incubated at 37°C and 5% CO_2_ for 1 h. After the incubation, 25 μl of CellTiter 96, AQueous One Solution Cell Proliferation Assay (MTS) (Promega, Madison, WI, USA) was added to the cell culture medium, and incubated at 37°C for 1 h. The absorbance of wells at 490 nm was measured with a microplate reader (SpectraMax M, Molecular Device, San Jose, CA, USA).

### Statistical Analysis

**Figure 1:**
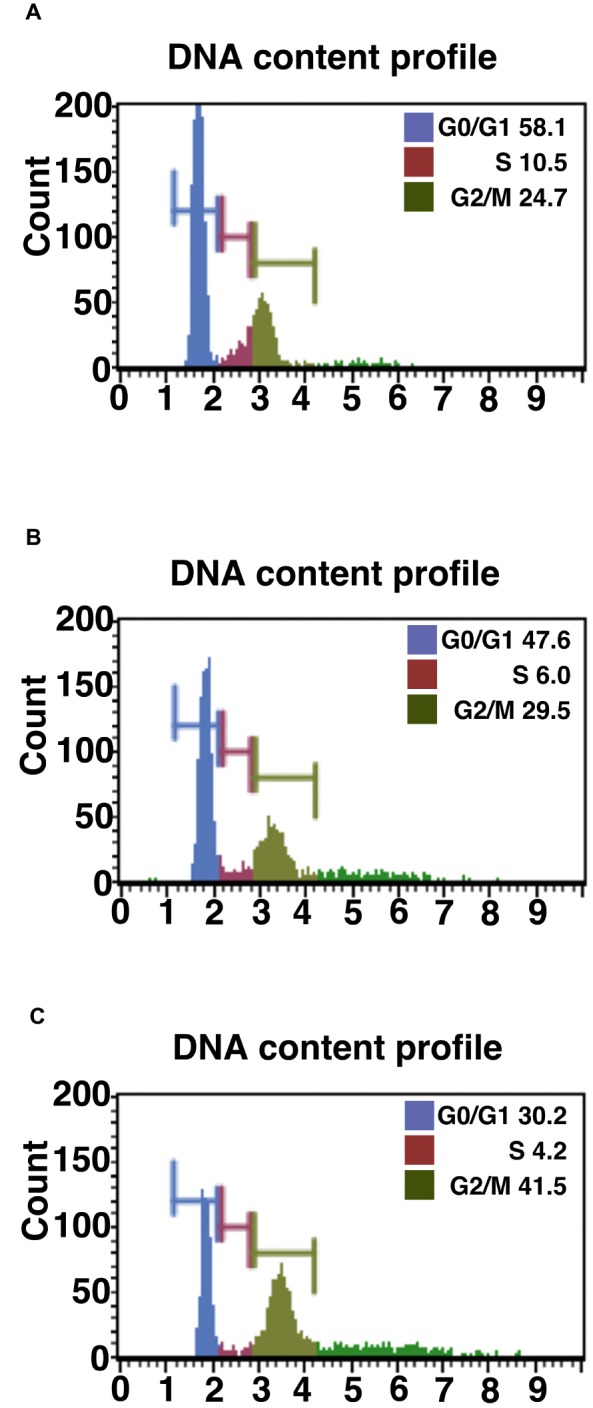
Cell cycle analysis of primary, K4D, and K4DT cells. **(A)** Cell cycle analysis of primary cells. **(B)** Cell cycle analysis of K4D cells. **(C)** Cell cycle analysis of K4DT + T cells.

**Figure 2:**
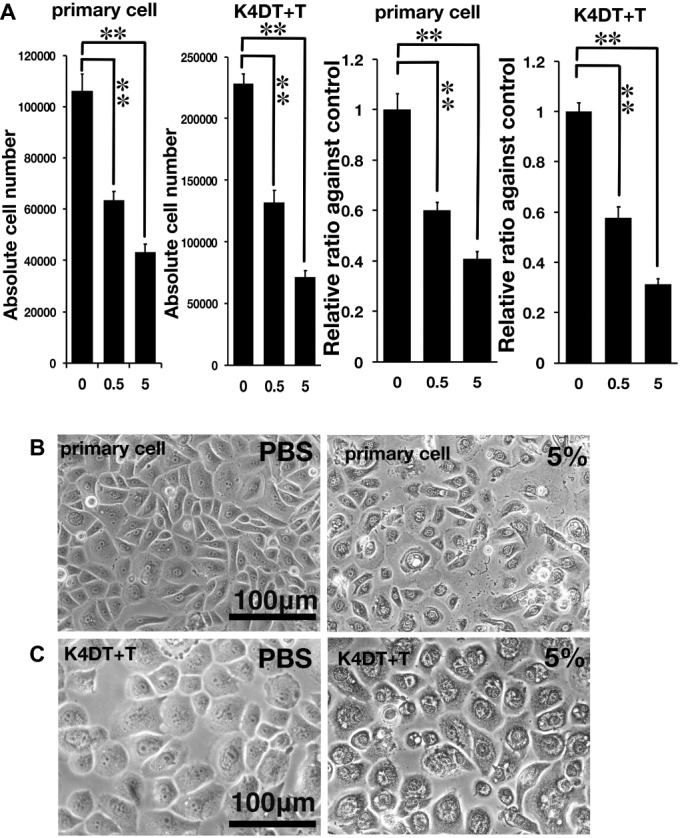
Assessment of glycolic acid (0.5 and 5%) as an irritant in primary and K4DT + T cells using the STE method. **(A)** The number of primary and K4DT cells. The data are expressed as the mean, with the error bars representing the standard error. Two stars indicate a statistical significance of more than 1%. **(B)** Cell morphology of PBS-treated (control) primary cells (left panel) and 5% glycolic acid-treated primary cells (right panel). **(C)** Cell morphology of PBS-treated K4DT + T cells (left panel) and 5% glycolic acid-treated K4DT + T cells (right panel).

**Figure 3:**
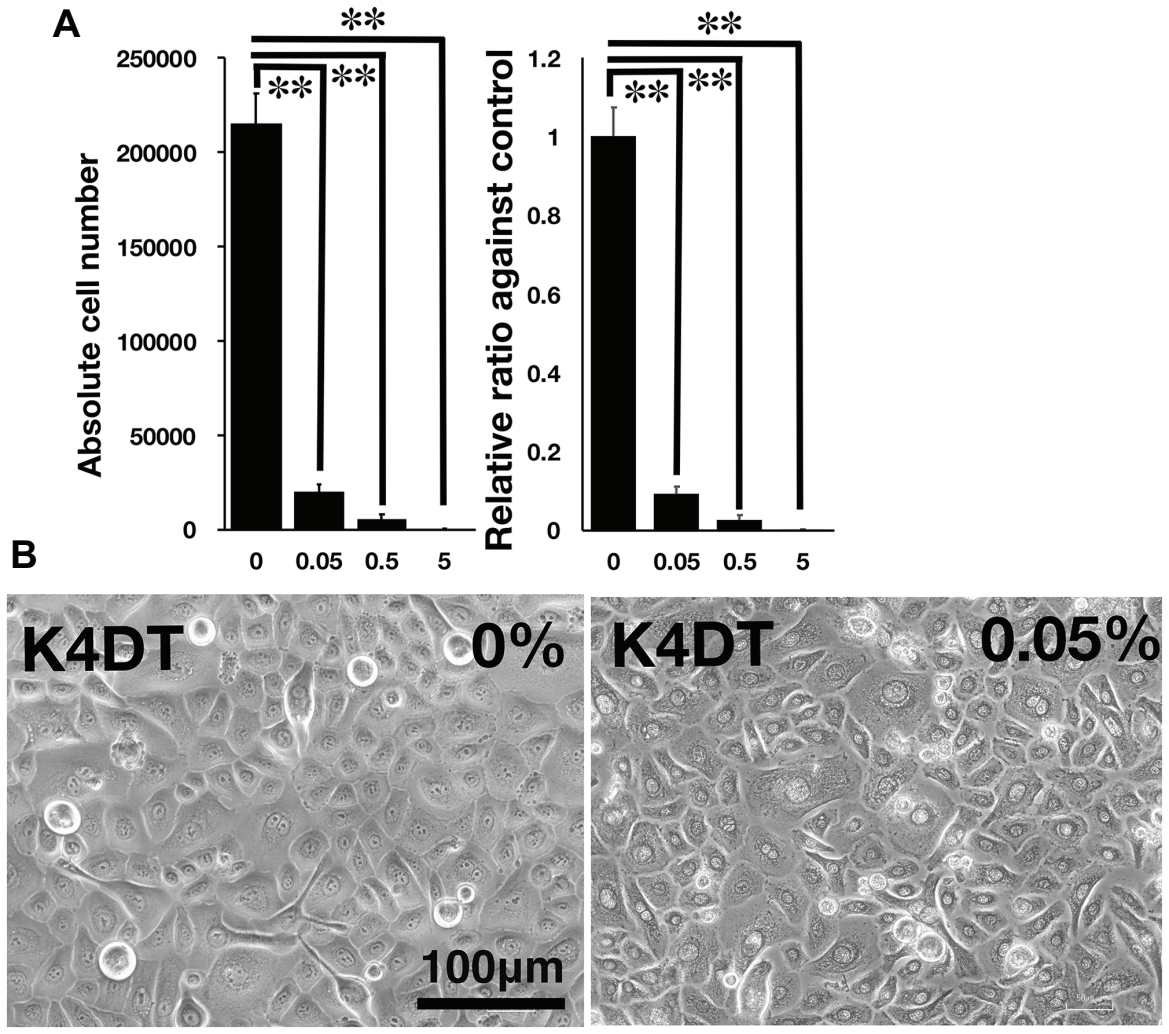
Detection of toxicity of Benzalkonium chloride using the STE method. **(A)** The number of K4DT cells. The data are expressed as the mean, with the error bars representing the standard error. Two stars indicate a statistical significance of more than 1%. **(B)** Cell morphology of PBS-treated (control) K4DT cells (left panel) and 0.05% Benzalkonium chloride-treated K4DT cells (right panel).

**Figure 4:**
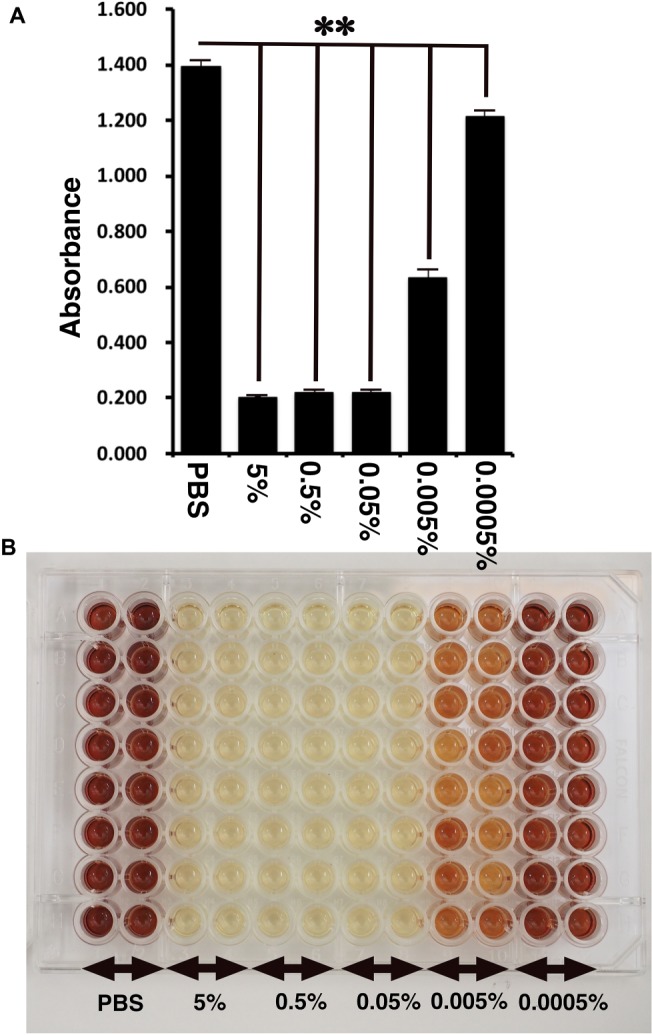
Detection of toxicity of Benzalkonium chloride using MTS assay. **(A)** The absorbance of K4DT cells. The data are expressed as the mean, with the error bars representing the standard error (*n* = 16). Two stars indicate a statistical significance of more than 1%. **(B)** The appearance of 96 microplates after 1-h incubation at 37°C after the addition of MTS reagent.

The data on cell cycle analysis (*n* = 6, Figure 1), absolute cell number after the STE method (*n* = 6, Figure 2, *n* = 8, Figure 3), and absorbance of MTS1 assay (*n* = 16, Figure 4) were analyzed with non-parametric Steel method using Statcell 3 (OMS Publishing, Tokyo, Japan).

## Results

### Morphological Changes After Gene Transduction

**Figure 5:**
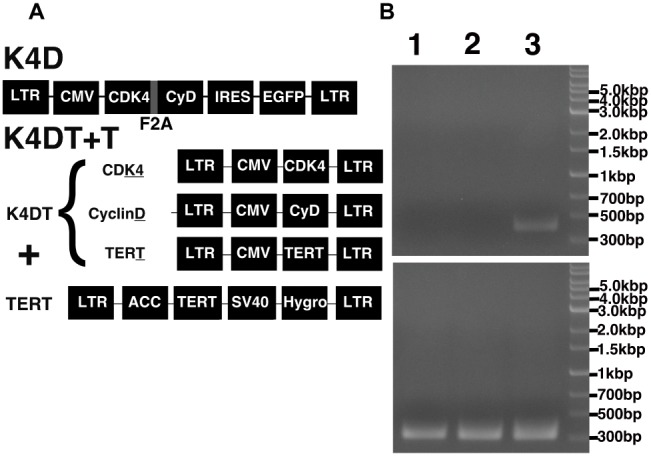
Schematic representation of the different expression vectors used in this study and confirmation of genomic integration and protein expression. **(A)** Structure of the expression vectors used to establish the K4D and K4DT + T cell lines. LTR, long terminal repeat; CMV, cytomegalovirus promoter; Hygro, Hygromycin-resistant gene. **(B)** Confirmation of the genomic integration of the TERT expression cassette by PCR: (1) primary cells; (2) K4D cells; (3) K4DT + T cells. The upper panel shows the specific 500-bp PCR product confirming the presence of the TERT expression cassette, whereas the lower panel shows the 400-bp PCR product derived from the human Tuberous Sclerosis Type II gene.

**Figure 6:**
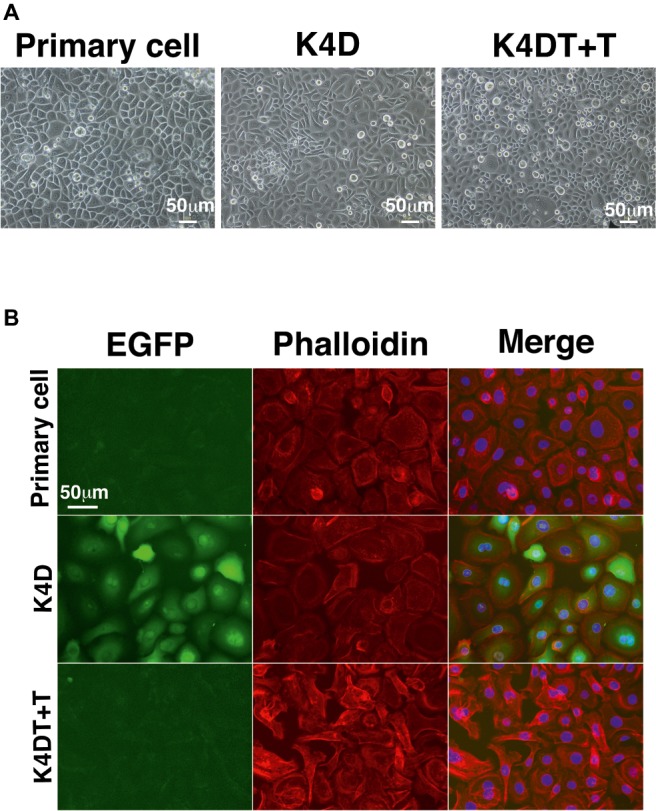
Cell morphology of primary, K4D, and K4DT + T cells. **(A)** Cell morphology using phase contrast microscopy at passage 0. **(B)** Morphological observation of the cytoskeleton (F-actin) and the nucleus, following staining with rhodamine-labeled phalloidin and DAPI, respectively. Left panel: EGFP fluorescence. Middle panel: phalloidin staining. Right panel: merged images of the phalloidin, DAPI, and EGFP stains.

To establish a corneal epithelial-derived cell line, we used the polycistronic lentivirus (CDK4R24CF2A-CyclinD) or a combination of the monocistronic lentiviruses expressing the R24 mutant of *CDK4, Cyclin D1*, and *TERT* (Figure 5A) to infect these cells. To evaluate the potential toxicity of these genes in the corneal epithelial cells, we compared the cell morphologies of the recombinant cells with that of primary cells. The recombinant cells had a similar morphology to primary cells (Figure 6A). Furthermore, the staining patterns of F-actin, stained with rhodamine-labeled phalloidin, in the recombinant K4D and K4DT + T cells were almost identical to that in the primary cells (Figure 6B), indicating that the expression of mutant CDK4, Cyclin D, and TERT do not affect F-actin distribution. From these data, we conclude that the exogenous expression of mutant CDK4, Cyclin D, and TERT do not change the cell morphology of corneal epithelial cells. Although the cell size was relatively smaller in K4D and K4DT cells, the smaller cell size was explained by the increased cell proliferation and constant cytoplastic replication speed (Cooper, 2004).

### The Detection of Transgenes by PCR and Western Blotting

**Figure 7:**
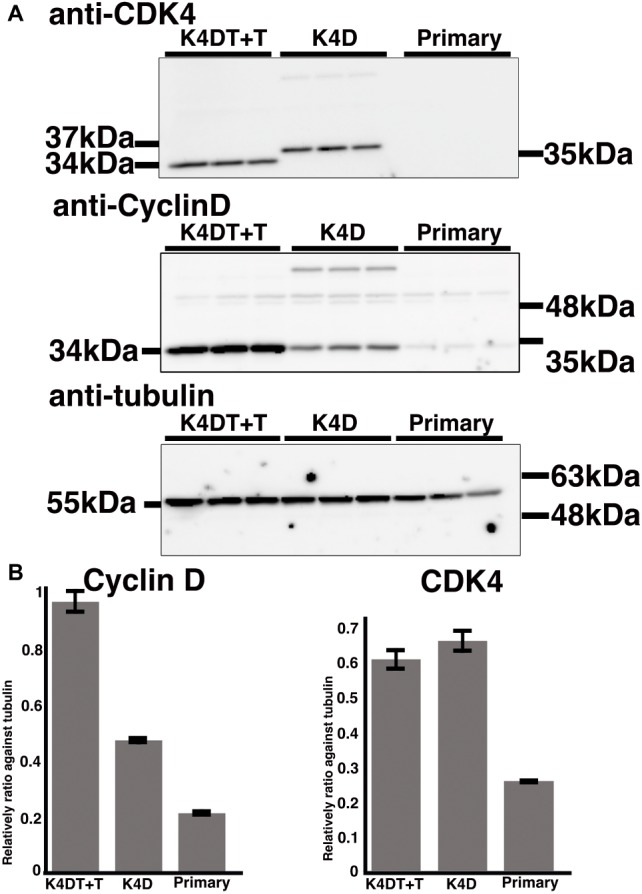
Detection of introduced proteins with western blotting. **(A)** Primary, K4D, and K4DT+T cells were applied into the western blotting using anti-CDK4, anti-Cyclin D1, anti-tubulin antibodies. The triplicated technical replicates were examined. **(B)** Quantitation results of the signal intensity with Image J software. The signal intensity of primary, K4D, and K4DT cells were shown in the graphs.

To monitor genomic insertion of exogenous genes, we performed PCR using genomic DNA extracted from primary, K4D, and K4DT + T cells (Figure 5B). The TERT gene was only detected in the genomic DNA isolated from K4DT + T cells, but not from genomic DNA isolated from primary or K4D cells. Furthermore, we confirmed the protein expression of Cyclin D and CDK4 by Western blotting (Figure 7). It should be noted that the Western blot data for the K4D recombinant cells showed a mobility shift for CDK4 to a higher molecular mass compared to CDK4 present in primary cells (Figure 7A). This observation is reasonable since the 2A peptide was inserted between the mutant CDK4 and Cyclin D, after which cleavage of the 2A peptide occurs at its carboxyl-terminal side, resulting in a shift in the mobility of the transgenic CDK4 (Figure 7A, top panel). From these results, we conclude that the genomic integration of mutant CDK4, Cyclin D, and TERT had been successfully achieved in corneal epithelial cells. Furthermore, from the band intensity of the Western blots, we concluded that the protein expression of CDK4 and Cyclin D was elevated in K4D and K4DT + T cells (Figure 7B).

### The Sequential Culture of Recombinant and Primary Corneal Epithelial Cells

**Figure 8:**
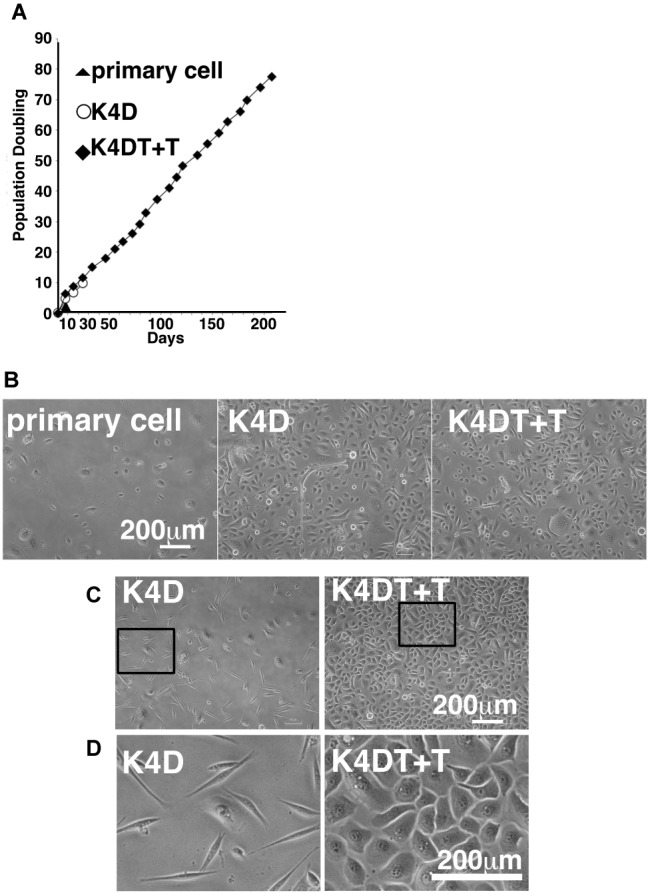
Cell growth status and morphological observation of primary, K4D, and K4DT + T cells. **(A)** Cell growth and sequential passaging of primary, K4D, and K4DT + T cell. Cell growth is represented by the cumulative population doubling value. **(B)** Morphological observation at passage 1 in the population doubling assay. **(C)** Cell morphology of K4D and K4DT + T cells at passage 3. Lower magnification of K4D (left panels) and K4DT + T cells (right panels). **(D)** Higher magnification of K4D cells (left panels) and K4DT + T cell (right panels).

We carried out sequential cell passaging to evaluate the cell proliferation of primary, K4D, and K4DT + T cells. At passage 1, the K4D and K4DT+T cells continued to proliferate, whereas the growth of the primary cells was completely arrested (Figure 8A). This cell cycle arrest in primary cells was reasonable since their cell proliferation was only guaranteed for five passages after thawing of the primary cells (based on the protocol provided by the supplier, the primary cells were already at passage 4 at the beginning of the study). The cell growth data were evaluated using the PDL, which showed the cumulative cell division number over sequential passages (Figure 8A). The data showed that cell proliferation in the K4D and K4DT + T cells was accelerated compared to primary cells (Figure 8B). Notably, the cell proliferation was continued more than 200 days in case of K4DT + T cell (Figure 8A).

Furthermore, we also observed that the K4D-expressing corneal epithelial cells had a morphology change around passage 3 that caused them to resemble fibroblasts (Figures 8C,D, left side). This morphological change was quite reproducible, being observed in four independent experiments. The ratio of fibroblast-like cells also gradually increased with increasing passage number.

### Accelerated Cell Growth in K4DT + T Corneal Epithelium Cells

We evaluated the effect of the expression of mutant CDK4, Cyclin D, and TERT on the cell cycle at passage 1. Figures 1A–C show representative results from this cell cycle analysis. The ratio of cells at each stage of the cell cycle is shown in different colors. In both the K4D and K4DT + T cells, there was a significant increase in the ratio of cells at G2/M and a decrease in the ratio of cells at G0/G1 (Table 1). From these data, we conclude that cell proliferation is accelerated after the expression of mutant CDK4 and Cyclin D.

**Table 1:** Cell cycle analysis of wild-type, K4D, and K4DT + T human-derived corneal epithelial cells.

	Cell cycle phase			
	G0/G1	S	G2/M	Debris
WT	58.2 ± 3.1	10.9 ± 1.0	22.5 ± 2.0	53.4 ± 4.7
K4D	48.8 ± 0.9^*^	6.6 ± 0.5^*^	27.5 ± 0.8^*^	56.4 ± 24.9
K4DT + T	29.0 ± 0.6^**^	4.9 ± 0.3^**^	40.0 ± 0.7^**^	51.1 ± 2.7

* *Significance at 5% level between WT and K4D, WT and K4DT + T*.

** *Statistical significance at 1%*.

### Lower Transgene Expression Levels in the K4D Fibroblast-Like Cells

**Figure 9:**
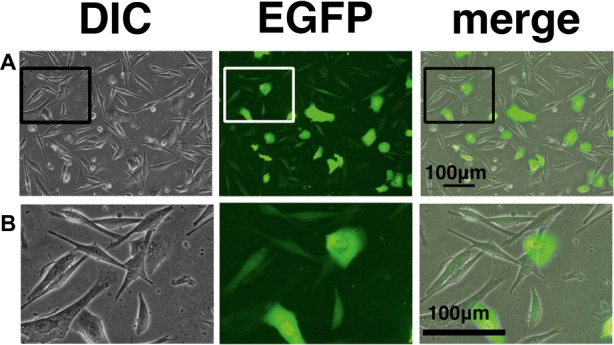
Cell morphology and EGFP expression of K4D cells at passage 3. **(A)** K4D cells at passage 3. Cell morphology by differential interference contrast microscopy (DIC, left panel), EGFP fluorescence (EGFP, middle panel), and merged images (merge, right panel). **(B)** High-magnification view of K4D cells at passage 3 by DIC (left panel), EGFP fluorescence (EGFP, middle panel), and merged images (right panel).

**Figure 10:**
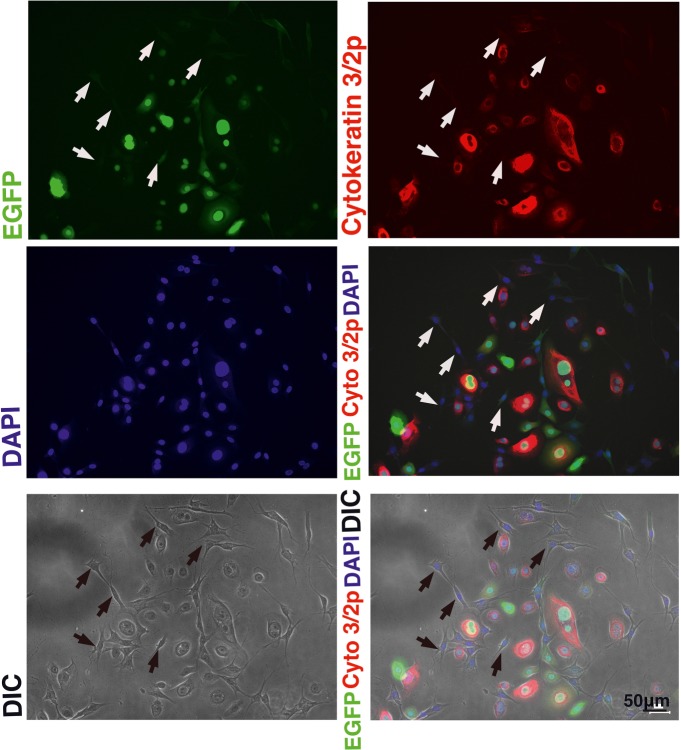
Immunostaining of K4D cells expressing EGFP. (Upper left) EGFP expression in K4D cells at passage 3. (Upper right) Immunostaining of cytokeratin 3/2p in K4D cells detected with Alexa 568. (Middle left) Nuclear counterstaining with DAPI. (Middle right) The merged picture among EGFP, cytokeratin 3/2p, and DAPI channels. (Lower left) Cell morphology of difference in contrast (DIC). (Lower right) The merged picture among EGFP, cytokeratin 3/2p, DAPI, and DIC channels. Note that fibroblast-like cells that are indicated by arrows were neither negative for EGFP and cytokeratin 3/2p.

We wondered why the fibroblast-like cells appeared in culture of the K4D cells from around passage 3 and onwards. To obtain a clue to explain this phenomenon, we examined the cells by fluorescence microscopy, as shown in Figures 9A,B. Interestingly, the fluorescence intensity in the fibroblast-like cells was much lower than that in cells with an epidermal-like shape. The expression cassette for CDK4-F2A-CyclinD is linked with EGFP *via* an IRES sequence. Therefore, the protein levels of EGFP should be correlated with that of CDK4-F2A-Cyclin D. Since the percentage of fibroblast-like cells increased after several passages, we conclude that the K4D corneal epithelial cells without TERT generated using the polycistronic expression vector are not suitable for use as a cell line for *in vitro* irritant testing. To evaluate the biological status of the fibroblast-like cells, we carried out the immunostaining of Cytokeratin 3/2p, which is the marker gene of the corneal epithelial cells. As shown in Figure 10, fibroblast-like cells (indicated with arrows in Figure 10) showed negative neither for EGFP nor for Cytokeratin 3/2p. From these data, the fibroblast-like cells have lost the nature of the epithelial origin cells.

### Irritation Test Using the STE Method of Glycolic Acid in Human Corneal Epithelial Cells *in vitro*

As described in the section “Introduction,” chemical irritants can be detected using the well-established STE method in rabbit-derived corneal epithelial cells (Takahashi et al., 2008). Accordingly, we evaluated the effect of a positive control irritant, glycolic acid, in human-derived corneal epithelial cells. We exposed either primary or K4DT + T human corneal epithelial cells to 0.5 and 5% glycolic acid. As shown in Figure 2A, the number of primary cells exposed to 0.5 and 5% glycolic acid solution was significantly decreased (Figure 2A, left side, primary cell) compared with the PBS control. Exposure of the K4DT + T cells to 0.5 and 5% glycolic acid also resulted in a statistically lower number of cells (Figure 2A). Decreased cell viability following exposure to 0.5 and 5% glycolic acid solution was reproduced in K4DT + T cells. Furthermore, we also compared the cell morphology between control and 5% glycolic acid-treated primary and K4DT + T cells before Accutase digestion. As shown in Figure 2B (right panel), cytoplasmic vacuolization and degeneration of the cell nucleus were evident in cells treated with 5% glycolic acid, indicating that the glycolic acid irritant severely damaged the cells. From these data, we conclude that our established K4DT + T human corneal epithelial cells have a potential use in evaluating chemical irritants.

### Irritation Test of Benzalkonium Chloride in Human Corneal Epithelial Cells *in vitro*

We furthermore tested the toxicity of Benzalkonium chloride with the STE method using our established immortalized human corneal epithelial cells. The toxicity of Benzalkonium chloride to corneal epithelial cells is well recognized since Benzalkonium chloride is the primary preservative chemical additive for eye drops. As shown in Figures 3A,B, the STE method showed a significant reduction in cell number in all doses of Benzalkonium chloride. Furthermore, the cell morphology treated by Benzalkonium chloride showed the nature of the cellular membrane, which possibly resulted in the cell death of epithelial cells (Figure 3B).

### Cell Toxicity of Benzalkonium Chloride Detected by MTS Assay

CellTiter 96 AQueous One Solution Cell Proliferation Assay is a colorimetric method for determining the number of viable cells in proliferation, cytotoxicity, or chemosensitivity assays, which was provided by Promega. We treated the immortalized cells with PBS, 5, 0.5, 0.05, 0.005, and 0.0005% Benzalkonium chloride in PBS. As shown in Figure 4A, the living cell number can be detected as the absorbance of microplate detector at 490 nm. Interestingly, treatment of Benzalkonium chloride even at 0.0005% showed statistically significant reduction of the cell viability. The appearance of the treated plate is shown in Figure 4B. From these data, we concluded that our established corneal epithelial cell is a useful tool to detect eye toxicity.

## Discussion

In this study, we established a new human corneal epithelial-derived cell line through the co-expression of a mutant CDK4, Cyclin D, and TERT. An analysis of cell proliferation over sequential passages showed that primary cells ceased proliferating when grown at low cell densities. However, the K4DT + T cell lines showed enhanced cell growth even at low cell densities. This property should provide an advantage to our K4DT + T cell line since it increases the ease by which the cells can be handled and should increase the reproducibility of the toxicity test. In contrast, in primary cells, there were significant differences between different cultures. With respect to the K4DT + T cells, although we have confirmed cell proliferation up to a PD of 200 days, we believe that the cell line is sufficiently well established that it can be shared with toxicology scientists.

When cells are exposed to cellular stresses, such as low cell density, the stressed cells accumulate the p16 protein, which is a negative regulator of the cell cycle (Meerson et al., 2004). The p16 protein binds to CDK4, resulting in the inactivation of the CDK4-Cyclin D enzymatic complex and a halt in cell proliferation. In cells expressing the R24C mutant CDK4, the p16 protein cannot bind to the mutant CDK4 protein, since the R24C mutation changes the protein structure of the binding pocket for p16. Co-expression of Cyclin D, therefore, induces constitutive activation of the mutant CDK4-Cyclin D complex, and this mutant CDK4-Cyclin D complex induces the phosphorylation of the tumor suppressor protein, retinoblastoma protein (RB), resulting in increased cell proliferation (Fukuda et al., 2018). Since this inactivation is limited to the RB pathway, the function of p53, which can be viewed as the guardian angel of the genome, remains intact. Although the expression of mutant CDK4 and Cyclin D accelerates cell proliferation, cells co-expressing these two proteins cannot escape from the shortening of the telomere sequence found at the end of chromosomes. To overcome this limitation, the additional co-expression of TERT allows for an extension of the telomere sequence, essentially creating immortalized cells. Since we confirmed the integration of the TERT cassette in the KD4T + T cells (Figure 5B), these cells have the potential to proliferate indefinitely.

Interestingly, corneal epithelial cells only expressing the mutant CDK4 and cyclin D showed evidence of a cellular morphological change from an epidermal shape to a fibroblast-like shape (Figures 9A,B). Two possibilities could explain this morphological change in these cells. The first possibility is the lack of the TERT gene and shortening of telomere repeat. Due to the lack of TERT, the epithelial might limit the cell proliferation due to the shortening of the telomere repeat sequence at the end of the chromosome. The second possibility is the decreased transcriptional activity of the cytomegalovirus (CMV) promoter due to a lack of TERT expression. Although the detailed molecular mechanism is unknown, we had observed that the protein levels of mutant CDK4 and Cyclin D, driven from the CMV promoter, are significantly elevated when we co-expressed TERT in megabat-derived cells (Fukuda et al., unpublished data). As supportive evidence for this notion, we detected that fibroblast-like cells were negative neither for EGFP nor for Cytokeratin 3, which is the marker gene for corneal epithelial cells (Figure 10). These data indicate that fibroblast-like cells already lost the nature of corneal epithelial cells.

After establishing the new human corneal epithelial cell line, we carried out an irritation toxicity test, comparing both primary and K4DT + T human corneal epithelial cells. As a first step, we evaluated glycolic acid as an irritant, assessing its cell toxicity *in vitro*. Exposure to glycolic acid resulted in a significant, dose-dependent decrease in cell viability. The K4DT + T cells showed a significant decrease in cell viability at both doses of glycolic acid (0.5 and 5%). Our data suggest that the human corneal epithelial K4DT + T cells have the potential to detect chemical irritants. Although the original STE method was developed using rabbit-derived corneal epithelial cells, evaluations using human-derived cells should increase the precision of the toxicology test.

Furthermore, we evaluated the toxicity of Benzalkonium chloride with the STE method. As shown in Figure 2, Benzalkonium chloride showed strong toxicity against our immortalized corneal epithelial cell. Based on the results of STE method, we further tested the toxicity of Benzalkonium chloride at PBS, 5, 0.5, 0.05, 0.005, and 0.0005% with MTS assay. In principle, MTS assay is a colorimetric assay for assessing cell metabolic activity. NAD(P)H-dependent cellular oxidoreductase enzymes are capable of reducing the tetrazolium dye MTT 3-(4,5-dimethylthiazol-2-yl)-2,5-diphenyltetrazolium bromide to its insoluble formazan, which has a purple color. Therefore, cell viability can be detected by the absorbance at 490 nm. As shown in Figure 4, notably, we detected the cytotoxicity even at the 0.0005% Benzalkonium chloride solution. These results indicate that our established cells are quite sensitive to the toxicity of the chemicals.

Recently, we have also succeeded in generating corneal epithelial-derived cells that express a secreted form of luciferase (Goko et al., unpublished data). Since the sensitivity of luciferase detection is quite high, we can use this to estimate the number of surviving cells after chemical treatment. The use of a secreted type of luciferase will allow us to monitor surviving cells in the culture medium, which should allow for use in high-throughput screening. Our established cells should contribute to accuracy in the evaluation of chemical toxicity and reduce the sacrifice of animals in experiments, which will be required for next-generation science.

## Author Contributions

TF, RG, TE, KT, and RS did the experiments. KN, ES, HT, and TF designed the experiments. TK contributed to essential experimental materials.

### Conflict of Interest Statement

The authors declare that the research was conducted in the absence of any commercial or financial relationships that could be construed as a potential conflict of interest.
